# Dry Bean Preferences and Attitudes among Midwest Hispanic and Non-Hispanic White Women

**DOI:** 10.3390/nu11010178

**Published:** 2019-01-15

**Authors:** Donna M. Winham, Megan E. Tisue, Shelly M. Palmer, Karen A. Cichy, Mack C. Shelley

**Affiliations:** 1 Food Science & Human Nutrition, Iowa State University, Ames, IA 50010, USA; metisue@iastate.edu (M.E.T.); spalmer@iastate.edu (S.M.P.); 2 USDA-ARS, Sugarbeet and Bean Research, Michigan State University, East Lansing, MI 48824, USA; Karen.Cichy@ars.usda.gov; 3 Political Science and Statistics, Iowa State University, Ames, IA 50010, USA; mshelley@iastate.edu

**Keywords:** dietary acculturation, consumer preferences, legumes, pulses, canned, poverty, attitudes, cooking time, food security, Mexican-Americans, immigrants, Latinas

## Abstract

Bean (*Phaseolus vulgaris* L.) intakes in the United States (US) lag behind dietary recommendations despite their positive nutrition profile, health benefits for reducing chronic disease risk, and inclusion in nutrition assistance programs. Low-income groups, including Hispanics, have an increased risk of cardiovascular disease, type 2 diabetes, obesity, and some cancers. Hispanic dietary quality and bean consumption may decline with increasing acculturation. Intakes at recommended levels could improve health in all vulnerable low-income populations. The study objectives were to describe dry and canned bean preferences, consumption frequency, and attitudes among low-income Hispanic and non-Hispanic white women, and to assess if these characteristics differed by ethnicity and acculturation level among the Latinas. A convenience sample of 158 women, aged 18–65 years, completed a written survey in English or Spanish at two healthcare clinics, one Special Supplemental Nutrition Program for Women, Infants and Children office, and five County Extension nutrition education and outreach programs in Iowa. Less acculturated Latinas consumed beans more often, preferred dry to canned, bought in bulk, valued color and shape in dry bean selection, and held less positive attitudes toward canned beans in contrast to bicultural/more acculturated and non-Hispanic white women. Ethnicity and acculturation level have a role in varying purchase patterns and attitudes regarding dry and canned beans. Culturally-held differences should be considered in nutrition programs and leveraged to increase consumption and improve health.

## 1. Introduction

Dry beans such as pinto, black, kidney, and navy (*Phaseolus vulgaris* L.) have been part of traditional and regional cuisines for centuries around the world [1]. Cultivated beans originated in two centers of domestication in Latin America, and their cultural integration is deep rooted [2,3]. In these areas, dry beans are purchased uncooked from the market, with fewer canned or other prepackaged options [2,3,4]. Consumption trends and patterns have been changing in Latin America in recent years as convenience foods are gaining popularity, yet beans remain an important cultural marker [4,5]. In contrast to Latin America, beans are not as prominent of a staple food in the US and are consumed at lower levels. Nationally, beans are readily available in supermarkets in many market classes and in both dry and canned forms [6]. Canned beans are often preferred by US consumers because of their convenience [5,6]. In the Midwest region, beans are familiar additions to casseroles, chili, stews, or Mexican foods, or served as a side dish [1].

Dry beans are beneficial for long term health of the human body, as well as the planet. The category of “pulses,” which includes beans, peas, lentils, and chickpeas, are vegetables with high protein, fiber, folate, vitamin E, iron, potassium, calcium, and magnesium [7]. Regular intake of pulses can improve dietary quality and is an excellent way to increase the number of vegetable servings consumed [7]. Daily amounts of 1/2 cup or less of beans have been associated with a lower risk of cardiovascular disease [8], reduction of total and low-density lipoprotein cholesterol [9,10], lower postprandial hyperglycemia [11], reduction of risk for some cancers [12], increased satiety and weight loss [13], and greater longevity [14,15]. They also support a sustainable food system through their nitrogen-fixation capacity which improves soil health and reduces the need for added fertilizer [16,17].

Federal nutrition programs that serve primarily low-income populations such as the Supplemental Nutrition Assistance Program (SNAP), National School Lunch Program, and the Expanded Food and Nutrition Education Program (EFNEP) promote beans and other pulses [18]. The Special Supplemental Nutrition Program for Women, Infants and Children (WIC) reaches over 7 million women and children each year [19]. Beginning in 2007, a policy change in WIC led to the inclusion of canned beans in food package options [20]. States now have flexibility in allowing dry and canned beans in the WIC food package to improve nutrition options, as well as to offer culturally sensitive options [21]. Use of canned foods saves meal preparation and cooking time that can be in short supply for limited-resource households [4]. National survey data indicate higher dietary quality for individuals who use canned vegetables [22,23]. Both dry and canned beans rank high on nutrient density, cost effectiveness, and acceptability [24]. Thus, canned bean use can aid in preserving the flavors of familiar cuisines while increasing food security and optimizing nutrition.

Recognizing the scientific evidence that dry bean consumption improves nutrition and promotes sustainable food systems, the 2015 Dietary Guidelines for Americans (DGA) Committee provides specific intake recommendations for dry beans and other pulses [25]. Despite the federal agency recommendation to include more beans in the diet, national survey data estimate women are consuming ½ cup and men 1 cup per week rather than the recommended 1½ to 2 cups per week [25]. In the 2011 Behavioral Risk Factor Surveillance Survey, almost 66% of adults reported eating beans at least 1 time per week. This frequency varied by ethnicity/race (77% Hispanics, 63% non-Hispanic whites (NHW), 52% non-Hispanic blacks) [26]. There is room for increasing bean consumption across the nation to improve nutrition and reduce disease risks [7,25].

Hispanics are twice as likely as NHWs to develop diet-related chronic conditions such as heart disease, type 2 diabetes, and obesity [27]. While they have higher intakes than other groups, bean consumption may be less than DGA recommendations and decline with further acculturation [7,28].

Hispanic classification is used to describe those with ancestral origins from a Spanish-speaking country [29]. The term masks the degree of Latino ethnic affiliation and the diversity of cultural experiences. Acculturation is the complex process of adopting or adapting sociocultural practices and beliefs of a different culture [30]. It is influenced by the proportion of other Latinos in neighborhoods, social networks, food environment, policies, immigrant characteristics such as education, age, gender, and other contextual factors [30,31]. Using acculturation level categories gives a finer-detail to the analysis and allows documentation of differences over the cultural change process [30,31]. Consumption patterns, attitudes, and preferences associated with acculturation are important to identify in variable settings since aggregated data can homogenize local and regional differences and population needs [30,32]. Retaining or increasing bean consumption—including canned—in the diet through culturally familiar, nutritious food can positively influence the health of all [7,28].

There is limited information on the attitudes of low-income women toward beans or other pulses they choose to obtain through nutrition assistance programs [28,33,34,35]. This lack of knowledge hinders agency ability to promote beans and other pulses for better nutrition and align client needs with DGA recommendations. Knowledge of perceived stigma, status, current nutrition guidelines, perceived qualities of healthfulness, and desirability of beans can be used to guide intervention strategies [28,33,34]. Nutrition education programs that consider these preexisting beliefs can leverage positive cultural practices and proactively address myths and misconceptions. The examination of attitudes toward beans is critical to grasp the cultural meaning and significance of their use, and to develop effective strategies to increase consumption. This information gives context to recommendations and allows for the tailoring of interventions to meet priority group needs [36].

In Iowa, the median age of Latinas is 22.8 years with a poverty rate of 18.5%, in contrast to 39.3 years for all Iowa women and a 13.1% overall poverty rate for females [37]. While comprising about 6% (*n* = 189,818) of Iowa’s population in 2018, Latinos are estimated to be 12.3% (*n* = 424,077) of the total state populace by 2050 [37]. Finding effective ways to maintain or improve nutrition quality and health for this growing population segment is particularly salient for young Latinas, who are often disproportionally poor, at nutritional risk, and lack access to services [27,37,38,39]. Furthermore, women can be powerful behavior change agents in their own right [40]. Women have the potential to transmit food knowledge, attitudes, and behaviors vertically as caregivers to children and their parents or other older adults. Horizontally, women’s influence extends to partners, friends, and coworkers [40,41]. These spheres of influence are true for all women regardless of income level or ethnicity.

The study purpose was to assess bean purchase preferences and attitudes about dry and canned beans among low-income Hispanic and NHW women in Iowa. We hypothesized that: (1) bean preferences, purchasing practices, and attitudes will differ among Latinas as acculturation increases and by ethnicity; and (2) attitudes regarding the consumption of canned beans will be more positive among NHW women and more-acculturated Latinas. Survey results on the knowledge of the health benefits of beans, self-efficacy, health risk factors, and preferred source of nutrition/health information in this sample were previously reported [42].

## 2. Materials and Methods

### 2.1. Study Design and Procedures

Low-income Hispanic and NHW women aged 18–65 were eligible to participate in this cross-sectional descriptive survey. Data were collected between June and November 2016. The sites were two healthcare clinics, one WIC clinic, and five County Extension nutrition education and outreach programs. Program eligibility for the low-income health clinics and WIC is typically 185% of the federal poverty guidelines, the amount of which varies depending on household composition [18]. Households consisting of two adults and two children were eligible for WIC services if their income fell below or equaled $44,863 annually or $3739 monthly in 2016 [43]. Women were not screened for study eligibility as program participation was considered proxy confirmation of low-income status.

Data collection took place in the waiting rooms at the clinics. Recruitment materials focused on general food choices and did not mention beans to avoid biasing the sample to only women who ate them. Interested women approached the research team directly at an information table. Women could choose from English or Spanish versions for the self-administered written survey. In the data collection sessions at County Extension programs, a trained bilingual staff member discussed the study at the beginning of preexisting nutrition education and outreach sessions. Women who wanted to participate stayed to complete the survey. At least one bilingual staff member was present at all survey sites with Hispanic clientele to explain the study and the consent form. Respondents received an insulated grocery bag valued at $5 and a recipe booklet as incentives. The study protocol was approved by the Institutional Review Board of Iowa State University (#16-239).

### 2.2. Survey Instruments and Measures

Demographic and household characteristics were collected using the EFNEP entry form [44]. These included: age, self-identification as Hispanic, race, household composition, education, marital status, monthly food expenditures (not including government benefits), total household income, and use of other federal nutrition programs. The 6-item Core Food Security Module (CFSM) responses classified households as high food security (0–1), low food security (2–4), or very low food security (5–6) [45]. The Bidimensional Acculturation Scale (BAS) was used to assess acculturation level for Latinas [31]. The BAS generates a score for Hispanic and English-dominant domains, which are used to classify respondents as: Hispanic-dominant (less acculturated), bicultural, or English-dominant (more acculturated). The BAS, EFNEP entry form, and CFSM were used verbatim from their published Spanish and English versions [31,44,45].

Based on literature review and our previous research, participants were asked how often they eat beans over a 1-month period [46], if they buy beans of any type (dry or canned), if they purchase dry beans, and if they buy canned beans. Regarding the purchase of dry beans, women identified their preferred packaging: small, medium, or large bags; loose; bulk beans; or a combination of these. Women were asked to describe which characteristics they looked for in purchasing dry beans and canned beans. Options for characteristics of both dry and canned beans included price, cultural or family tradition (i.e., always buy this type), perceived quality, taste, nutritional value, and brand name. Dry bean questions incorporated additional characteristics of color, shape, and cooking time [35,47,48]. Respondents were asked if they preferred dry beans grown in a specific country, and if they would be interested in buying dry beans grown locally in Iowa [47].

To evaluate participant attitudes and perceptions toward consuming any beans, a series of seven statements (do not like taste, family will not eat, cause intestinal gas, only poor people eat, take too long to cook, difficult to make foods with, not eaten by friends) were posed. Statements were modeled after a previous study in Arizona [35,49]. A second set of seven statements focused specifically on canned bean perceptions (do not taste good, family will not eat, if I cannot cook my own beans will go without, not true to culture, not healthy, too expensive, have preservatives) [35]. Question response options were: (1) strongly disagree, (2) disagree, (3) agree, or (4) strongly agree, and included a fifth option of “do not know”.

### 2.3. Data Analysis and Transformations

Data were entered and analyzed using SPSS Version 24 for Windows (IBM, SPSS, Armonk, NY, USA). All variables were examined via frequency distributions for missing data, and the explore command for identifying outliers, and confirming distribution normality for continuous measures. Univariate statistics and correlations between variables were examined for initial relationships. A probability level of *p* < 0.05 was used to indicate statistical significance. The Likert-type questions for dry bean and canned bean perceptions were analyzed separately for scale reliability. The 7-item bean attitude scale had a Cronbach’s alpha value of 0.78, indicating “acceptable” reliability. Canned bean attitude responses were mostly phrased in the negative. These were reverse-coded prior to summation so that a higher scale value would reflect positive attitudes toward canned beans. The 7-item canned bean attitude scale had a Cronbach’s alpha of 0.82, signifying “good” reliability [50]. Analysis of variance was used to check for differences in attitude scale means by acculturation categories. Predictive models of dry and canned bean attitude scales were developed using a general linear model.

## 3. Results

### 3.1. Demographics, Household Characteristics, and Food Security

Table 1 shows the demographic and household characteristics of the 158 women who completed the study by acculturation and race. The survey completion rate was 87% (158/182). Fifty-eight percent (92/158) of the women stated they were Hispanic. Within this group, 77% identified as Mexican, 10% Central American, and 13% other Latina ancestry. Based on the BAS, there were 39 Hispanic-dominant, 45 bicultural, 8 English-dominant Latinas, and 66 NHW women. The English-dominant Latinas were analyzed together with the bicultural women because their mutual demographic characteristics were more similar than to the NHW women. The mean age of all women was 36.4 ± 12.7 years. Bicultural/English-dominant Latinas were significantly younger than the Hispanic-dominant and NHW women (*p* = 0.001). Most Hispanic-dominant women were married, had more children under age 18 (*p* = 0.003), and more adults in the household size in comparison to their peers (*p* < 0.016). They spent more money on food each month (*p* = 0.008) and had lower education (*p* < 0.001) than the bicultural/English-dominant or NHW women. Over 43% of the Hispanic-dominant Latinas reported using child nutrition programs (i.e., National School Lunch Program) (*p* < 0.001). The NHW women were the most food insecure (*p* = 0.003) and had the highest percentage of SNAP benefits use (33% vs. ~10% for all Latinas) (*p* = 0.001).

### 3.2. Consumption Frequency and Purchasing Preferences for Dry and Canned Beans

Bean consumption frequencies, purchasing practices, and preferred characteristics of beans by acculturation status among respondents are shown in Table 2. Fewer than 11% of the respondents ate beans five or more times per week, an approximation of the 2015 DGA recommendation. Bean consumption frequencies were highest for Hispanic-dominant and bicultural/English-dominant women at 3–4 times per week (38.5%; 30.2%), while a plurality of NHW women ate beans 2–3 times per month (37.9%) (*p* < 0.001). 

All Hispanic-dominant and 90.6% of bicultural/English-dominant women reported purchasing beans of any type (dry or canned), in contrast to 72.7% of NHW women (*p* < 0.001). For bean form, 15.2% did not buy any beans, 18.4% only bought dry, 16.5% only bought canned, and 50% bought both (*p* = 0.011). Purchasing type was not significantly different within acculturation group for the Hispanic-dominant (0% did not buy beans, 48.7% dry only, 5.1% canned only, 46.2% dry and canned) or bicultural/English-dominant women (9.4% did not buy beans, 17.0% dry only, 17.0% canned only, 56.6% dry and canned). Dry vs. canned bean purchasing was significantly different for the NHW women (28.8% did not buy beans, 1.5% dry only, 22.7% canned only, 47.0% dry and canned; *p* < 0.001; data not shown in table).

Significantly fewer bicultural/English-dominant Latinas (73.6%) and NHW women (48.5%) bought dry beans in comparison to Hispanic-dominant women (94.9%; *p* < 0.001). Of the 68% (108/158) who reported purchasing dry beans, women preferred small 1-pound bags (32.4%) or a combination of loose and bagged (28.7%). Hispanic-dominant women preferred a combination of loose and bagged (32.4%). Bicultural/English-dominant women preferred medium bags (36.1%), and NHW women (48.6%) preferred small bags (*p* = 0.026). Respondents were asked to select all that apply for nine dry bean characteristics. Price was the most selected characteristic of interest for all women, followed by taste. More Hispanic-dominant women were interested in the color (*p* = 0.001), and shape (*p* = 0.002) of dry beans than their peers. About 25% of the Hispanic-dominant and NHW women selected dry beans that cooked quickly in in contrast to 5.1% of the bicultural/English dominant women *p* = 0.038). (Other dry bean traits were not significantly different by BAS categories. For 18–46% of the respondents, price—46%, tradition—34%, quality—35%, taste—28%, nutritional value—22%, and brand—19% were indicated as important selection criteria.

Almost 91% of all women who bought dry beans did not prefer a specific country of origin for them. However, 11.8% of Hispanic-dominant and 7.7% of bicultural/English-dominant women favored beans from Mexico or Central America. About 6% of NHW women preferred US grown dry beans (not significant). Sixty-three percent of NHW women were interested in Iowa dry beans, compared to 29.4% of Hispanic-dominant and 31.6% of bicultural/English-dominant women (*p* = 0.002).

Canned beans were purchased by 66.5% (105/158) of the women. Bicultural/English-dominant women had the highest percentage (74.6%) of canned bean purchases, followed by NHW women (68.8%), and Hispanic-dominant women (51.1%) the lowest (*p* = 0.018). Respondents were asked to select all that apply for six canned bean characteristics. Forty-one percent of all women stated taste was central to their selection of canned beans. Significantly more NHW women were interested in canned bean price (58.7%) than were the bicultural/English-dominant (41.0%) or Hispanic-dominant women (20.0%) (*p* = 0.012). Bicultural/English-dominant (30.8%) and Hispanic-dominant (20.0%) women chose a canned bean type based on tradition more than NHW women (8.7%; *p* = 0.035).

Almost half of the women who bought canned beans (49%) did not buy a specific brand or could not recall the brand name. For the 51% of women who provided a canned bean name, the most frequently mentioned ones were generic or store brand (14.3%), Bush (8.6%), La Costeña (8.6%), Goya (7.6%), La Preferida (6.7%), or a mix of other brand names (5.6%). Ninety-four percent of all women purchased their groceries from supermarkets. About 4% reported using ethnic food stores such as *tiendas*, and 2% stated they shopped at warehouse stores. Store choice was not significant by ethnicity or acculturation categories (data not shown).

Responses to seven statements on attitudes toward beans are shown in Table 3. About 17% of all respondents agreed or strongly agreed that they did not like the taste of beans. Eighty-nine percent disagreed that only poor people eat beans. About 49% agreed or strongly agreed that dry beans take too long to cook. Seventy-eight percent agreed that their friends ate beans and 16.6% stated they did not know. Forty-six percent of all women agreed or strongly agreed that beans cause intestinal gas. Almost 24% of Hispanic-dominant, 12% bicultural, and 7.7% of NHW women stated “do not know” regarding intestinal gas (non-significant trend *p* = 0.063). Three of the general bean statements were significantly different by BAS categories. Twenty-three percent of the NHW women agreed or strongly agreed that their families or children would not eat beans in comparison to about 5.9% of the bicultural/English-dominant women and 5.2% of their Hispanic-dominant peers (*p* = 0.041). About 8% of the NHW women agreed it was difficult to make food with beans whereas none of the Hispanic-dominant women and 3.8% of the bicultural/English-dominant Latinas strongly agreed or agreed with this statement (*p* = 0.033). The mean bean attitude scale was not significantly different by acculturation level. A generalized linear mixed model tested if five variables (acculturation level, bean consumption frequency, age, household size, and food security category) predicted the bean attitude scale. The results of the analysis indicated the corrected model explained 40.2% of the variance in the bean attitude scale (*R*^2^ = 0.402; *F* (36,124) = 1.64, *p* = 0.031; partial *η*^2^= 0.402; power = 0.984). Bean attitude scale was predicted by bean consumption frequency (*F*(4) = 4.83; *p* = 0.001; partial *η*^2^ = 0.180; power = 0.947).

Table 4 shows the percentage distributions of attitude statements about canned beans. Over 20% of all women agreed or strongly agreed that they would not eat beans if they could not cook themselves. Almost 49% agreed or strongly agreed canned beans contained preservatives, and 29.7% stated ‘do not know.’ Responses to five of the seven questions were significantly different by BAS categories. More Hispanic-dominant women agreed that canned beans do not taste good (*p* < 0.001), their family will not eat them (*p* < 0.001), were not true to their culture (*p* = 0.003) and are not healthy (*p* = 0.006) in comparison to the bicultural/English-dominant and NHW women. The mean canned bean attitude scale was higher (more positive) for NHW and bicultural/English-dominant women than for Hispanic-dominant women (*p* = 0.001). The same generalized linear mixed model was applied to the canned bean attitude scale as described above for the general bean attitude scale. The corrected model explained 41.2% of the variance in the canned bean attitude scale (*R^2^*= 0.412; *F* (36,124) = 1.71, *p* = 0.022; partial *η*^2^ = 0.412; power = 0.988). Acculturation level (*F*(2) = 3.091; *p* = 0.050; partial *η*^2^ = 0.066; power = 0.582), and food security category (*F*(2) = 3.250; *p* = 0.043; partial *η*^2^ = 0.069; power = 0.605) were also significant predictors.

## 4. Discussion

This research is one of the few studies to describe dry and canned bean purchase preferences and attitudes among low-income Latina and NHW women in the Midwestern US [28,35]. Our findings confirmed our first hypothesis by identifying significant differences by acculturation and ethnicity in bean consumption frequencies, preferred purchasing traits, and attitudes between Latinas and NHW women in Iowa. Bean consumption and purchasing of beans of any type as well as dry beans was higher among Latinas compared to NHW women and declined as acculturation increased. While almost all Latinas purchased dry beans, less than half of the NHW women did. It is possible the NHW women may need further education or awareness of the dry bean preparation process.

In a recent focus group study of general nutrition behaviors, low-income NHW and African American women in Iowa enrolled in WIC described that not knowing how to cook dry beans was a point of frustration [34]. In contrast, a survey of 131 mostly white WIC participants in a Florida county expressed confidence in preparing dry beans [33]. These variable reports may reflect regional differences in populations and WIC practices. At the national level, WIC has recently reduced the amount of pulse vouchers provided to program participants based on lower levels of redemption [21]. Iowa WIC stopped allowing canned beans in 2012, citing concerns with controlling costs. Better culinary education and outreach to consumers on how to prepare dry beans may be needed. For participants to better use the WIC bean allowance, specific cooking details on packaging, and beans that are consistent in their cooking time, could potentially increase consumption [34,51]. With over 7 million mothers and children in the WIC program alone, the potential to improve dietary quality, reduce disease risk, and shape healthy nutrition behaviors through bean consumption is great across all ethnicities served [19].

Our results support that US Latinas and NHW women are interested in specific dry bean characteristics and traits. Price was the top selection item for almost half of the women who buy dry beans regardless of ethnicity. About 44% of all women agreed or strongly agreed that dry beans took too long to prepare. Additionally, cooking time was an important criterion when buying dry beans for Hispanic-dominant Latinas and the NHW women. Lack of time for meal preparation has been reported a concern in other surveys [4]. Globally, fast cooking beans are preferred for convenience issues as among many of our participants [51,52,53,54]. The economic practicality of using less electricity or fuel can be an important factor in limited-resource households [55].

Both Hispanic-dominant and bicultural/English-dominant women were interested in bean color, and Hispanic-dominant women looked at shape in choosing dry beans. With their higher dry bean purchase rate, it is not surprising that Latinas were more concerned with these attributes than the NHW women. Since less than half of the NHW women purchased dry beans, the group may have less familiarity with how to identify some quality traits. In comparison, consumers in Mexico selected beans based on cooking time, color, taste, and flavor [2]. Preferred traits of dry beans were the greatest characteristic that influenced purchasing, and Mexican consumers held to their favorite bean even when offered lower cost alternatives [2]. Bean consumption remains high across all economic sectors at 11 kg (24 lbs) on average per capita in Mexico [3]. Similarly, in East Africa, where dry bean consumption is much higher than in the US, consumer preference relates strongly to seed color, shape, gravy quality (i.e., color and thickness), cooking time, and price [51,52,53,54]. Unlike in Mexico or East Africa, most US dry beans are prepackaged for sale. Consumers can select certain market classes, or brands, but may be limited in selecting color or shape. Further investigation on the selection processes for dry beans is necessary to confirm and meet marketing preference needs in the US.

Zamora and Bernsten found that Central American immigrants were interested in beans grown from their home countries [47]. The Iowa Latinas did not share this view as strongly. It is possible imported beans are more expensive in the Midwest, or alternatively, dry beans are relatively fresh since grown in adjacent states. For the majority of NHW women, there was a surprisingly strong interest in locally grown dry beans. Purchasing local foods enhances the regional economy and benefits the environment [56,57]. Increased interest in locally grown, non-genetically modified, and climate-smart crops is at a high nationally. Promoting local dry beans may be a leverage point for increasing low-income NHW women’s intakes of them [58]. Niche markets for black beans currently exist in Iowa [59]. Other pulse species like lima, black-eyed peas, mung, and adzuki have been grown successfully in Iowa. In addition, the growing Latino market in Iowa could support higher bean production [37,57]. Local pulse production could encourage diversification of Iowa’s agricultural landscape while promoting human and food system health [56,58].

Over half of the NHW women were food insecure, yet they consumed beans the least often. Although these women had positive attitudes toward dry and canned beans, they are underutilizing these nutrient-dense, cost-effective foods [7,24]. A similar pattern of high food insecurity and infrequent bean consumption among low-income NHW women in comparison to Hispanic-dominant and bicultural Latinas was observed in Arizona [35,48]. Budgeting, meal planning, and money management skills were reported by EFNEP participants in Massachusetts as effective ways to reduce food insecurity, and beans can play a greater role if messaging is reaching the targeted populations [60]. NHW women expressed concerns with household members not eating beans, and stated difficulty in making foods with them, but Latinas did not share these two challenges in the current survey. Only a few participants agreed that beans were “poor people’s” food which suggests the women in the current study do not view beans as stigmatized. In contrast, low-income NHW and African American women in Iowa focus groups were vocal about not liking the taste of beans and associating them with poverty and stigma. They also expressed concerns about feeding their families ‘cheap’ food, and that their male partners wanted meat in meals [34]. These issues were not explored in the current survey but suggest other sociocultural aspects to consider in future research.

Our second hypothesis, that attitudes toward canned beans would be more positive among NHW women and would increase with acculturation in Latinas, was confirmed. The negative view of canned beans may reflect a lack of understanding of the nutritional value of canned foods and how they are processed [24]. Similar negative attitudes toward canned beans were observed in two studies among less acculturated Latinas in Arizona [28,35]. In interviews with Hispanic immigrant women in New York City, Park et al. found they preferred foods that were fresh and unadulterated by processing or long periods of storage [61]. Cambodian and Brazilian immigrants in Massachusetts also focused on the freshness of their cultural foods [62]. Qualitative inquiry on the barriers to canned bean use may elicit other sociocultural reasons for their avoidance by Iowa Latinas [63].

Bicultural/English-dominant women were more closely in agreement with Hispanic-dominant women for preference to cook beans themselves, the perception that canned beans are not true to their culture, and the view that canned beans are unhealthy. However, some responses were more nuanced. Of the three groups, the bicultural/English-dominant women had the highest level of canned bean purchases. Their attitudes toward canned bean expense and family acceptability were more like those of the NHW women.

Concerns about preservatives in canned beans may stem from perceptions of equating high sodium content in with other chemicals [64]. Canned bean sodium content has decreased over the past 10 years. Additionally, nutrient comparisons are typically done on whole canned product including brine [65]. Estimated reduction in sodium is as high as 40% when they are drained and rinsed prior to use. Low-sodium canned options are also available at a similar price in most stores. Nutrition assistance programs should include information on low-sodium alternatives as well as advising rinsing the canned product prior to use to reduce sodium content. Education efforts should concentrate on information about food processing procedures and label reading. Florida WIC participants reported higher bean consumption when they used canned [33].

### Limitations and Strengths of Study

There are several study limitations. These data are from a cross-sectional convenience sample of Hispanic and NHW women who were participants at WIC, Extension nutrition programs, or health clinics in central Iowa. These results may not be applicable to Hispanics overall, men, or other low-income women who are or are not eligible for nutrition assistance. The self-administered instrument may have contained written questions that respondents did not understand, even though efforts were made to pilot test the survey instrument for comprehension among a population of similar origin, and a bicultural staff person was available for assistance with Spanish. Participants were not asked which types of beans (pinto, black, navy, etc.) or the serving size of the amounts they typically consumed. This information would have been useful to identify or patterns for market class purchases. Reasons behind food choices or selection of bean characteristics were not asked.

Despite these limitations, the study has several strengths. Notably, it is one of the few reports on bean consumption, purchasing preferences, and attitudes among low-income US women, and gives perspectives of those who do not consume beans. It is unique in comparing trends by acculturation status among Latinas in contrast to NHW women. Information on Midwest Latinas adds to the literature on regional and local variations in dietary acculturation.

## 5. Conclusions

In summary, our results support tailoring messaging to increase dry bean consumption among low-income women by acculturation level for Latinas and addressing concerns specific to NHWs. Beans are a healthy component of Latino and American cuisines. Understanding limited-resource women’s attitudes and perceptions related to beans may aid in reducing disparities in food access, increasing nutrition knowledge, and improving health. Consumer attitudes toward products determine consumption behavior more so than knowledge alone and play an important role in food consumption decisions [65]. Nutrition education and Extension programs should promote bean consumption for better health and encourage increased usage of beans among low-income women in Iowa of all ethnicities. Results from the current study can contribute to Extension and other programming to shape and inform lifestyle decisions. Less than optimal bean consumption is of concern for all Americans, not only Latinas and/or limited-resource women. Nutrition education programs that consider perceived qualities and proactively address the myths and misconceptions can foster positive cultural practices that include healthy, sustainable beans in dry or canned form.

## Figures and Tables

**Table 1:** Demographic and household characteristics of low-income Iowa women by ethnicity and acculturation (*n* = 158).

Characteristic	Total	Hispanic-Dominant 25% (39)	Bicultural-English-Dominant 33% (53)	Non-Hispanic White 42% (66)
	mean ± standard deviation (SD)			
Age in years **	36.4 ± 12.7	38.9 ± 11.5 ^a^	31.2 ± 12.4 ^b^	39.0 ± 12.4 ^a^
Total household size ***	3.8 ± 1.8	4.8 ± 1.4 ^a^	3.6 ± 1.8 ^b^	3.2 ± 1.6 ^b^
Children under age 18 **	1.4 ± 1.4	2.0 ± 1.4 ^a^	1.3 ± 1.3 ^b^	1.2 ± 1.3 ^b^
Number of adults *	2.3 ± 1.0	2.7 ± 1.0 ^a^	2.3 ± 1.0 ^a^	2.1 ± 0.9 ^b^
Monthly food expense $ **	430 ± 267	542 ± 290 ^a^	431 ± 253 ^b^	367 ± 247 ^b^
	%			
Years of Education ***				
6th grade or less	7	28.2 ^a^	0 ^b^	0 ^b^
7–11th grade—Junior High	14.6	46.2 ^a^	0 ^b^	7.6 ^c^
12th grade or certificate	21.5	10.3 ^a^	24.5 ^a^	25.8 ^a^
Some college or tech school	36.1	10.3 ^a^	39.6 ^b^	48.5 ^b^
Bachelor’s degree or more	20.9	5.1 ^a^	35.8 ^b^	18.2 ^a^
Marital Status ***				
Single	24.7	2.6 ^a^	39.6 ^b^	25.8 ^b^
Married	48.1	79.5 ^a^	23.7 ^b^	35.5 ^b^
Cohabitating	12.7	15.4 ^a^	11.4 ^a^	12.1 ^a^
Divorced/Widowed/Separated	14.6	2.6 ^a^	15.1 ^b^	21.2 ^b^
Food Security Status ^1,^**				
High	60.9	72.7 ^a^	68.1 ^a^	48.3 ^b^
Low	23.9	24.2 ^a^	25.5 ^a^	22.4 ^a^
Very low	15.2	3.0 ^a^	6.4 ^a^	29.3 ^b^
**Federal Program Usage**				
Food assistance (SNAP) **	19.6	10.3 ^a^	9.4 ^a^	33.3 ^b^
Child nutrition programs ***	19	43.6 ^a^	11.3 ^b^	10.6 ^b^
WIC ^†^	17.7	25.6	13.2	16.7
Head Start	5.1	7.7	5.7	3
Temporary Aid for Needy Families	8.2	7.7	3.8	12.1

^1^*n* = 138; * *p* < 0.05; ** *p* < 0.01; *** *p* < 0.001; Values with the same superscript letters are not significantly different; ^†^ Special Supplemental Food Program for Women, Infants and Children.

**Table 2:** Bean consumption frequencies, purchasing practices, and preferred characteristics of low-socioeconomic status Iowa women by ethnicity/race and acculturation categories (*n* = 158).

Survey Question	Total	Hispanic-Dominant 25% (39)	Bicultural/English-Dominant 33% (53)	Non-Hispanic White 42% (66)
Bean consumption frequency ***				
Once per month or less	19.6	0 ^a^	17.0 ^b^	33.3 ^b^
2–3 times per month	27.8	17.9 ^a^	22.6 ^a,b^	37.9 ^b^
1–2 times per week	19	20.5 ^a^	20.8 ^a^	16.7 ^a^
3–4 times per week	22.8	38.5 ^a^	30.2 ^a^	7.6 ^b^
5 or more times per week	10.8	23.1 ^a^	9.4 ^a,b^	4.5 ^b^
Buys beans of any type ***				
Yes	85.4	100 ^a^	90.6 ^b^	72.7 ^c^
No	14.6	0 ^a^	9.4 ^b^	27.3 ^c^
Buys dry beans ***				
Yes	68.4	94.9 ^a^	73.6 ^b^	48.5 ^c^
No	31.6	5.1 ^a^	26.4 ^b^	51.5 ^c^
Buys canned beans? *				
Yes	65.7	51.1 ^a^	74.6 ^b^	68.8 ^b^
No	34.3	48.9 ^a^	25.4 ^b^	31.2 ^b^
**Of the 68% (108/158) who buy dry beans:**				
Preferred packaging *				
Small bags	32.4	21.6 ^a^	27.8 ^a,b^	48.6 ^b^
Medium bags	23.1	18.9 ^a^	36.1 ^a^	14.3 ^a^
Large bags	10.2	21.6 ^a^	8.3 ^a,b^	0 ^b^
Combination of loose and bags	28.7	32.4 ^a^	27.8 ^a^	25.7 ^a^
Did not answer	5.6	5.4 ^a^	0 ^a^	11.4 ^a^
**Preferred dry bean traits**				
Price	46.3	43.2	43.6	51.4
Tradition	34.3	43.2	38.5	20
Quality	35.2	43.2	33.3	28.6
Taste of beans	27.8	29.7	25.6	28.6
Color of beans **	22.2	40.5 ^a^	20.5 ^a^	3.1 ^b^
Nutritional value	22.2	16.2	25.6	25
Brand	19.4	21.6	23.1	12.5
Cook quickly *	17.6	24.3 ^a^	5.1 ^b^	25.0 ^a^
Shape **	13.9	29.7 ^a^	7.7 ^b^	3.1 ^b^
**Prefer to buy dry beans from a specific country? (*n* = 104)**				
No country preference	90.5	85.3	92.3	93.8
Yes, Mexico or Central America	6.7	11.8	7.7	0
Yes, USA or other	2.9	2.9	0	6.3
Interested in Iowa dry beans? **				
Yes	40.4	29.4 ^a^	31.6 ^a^	62.5 ^b^
Maybe	41.3	35.3 ^a,b^	55.3 ^b^	31.3 ^a^
No	18.3	35.3 ^a^	13.2 ^b^	6.3 ^b^
**Of 66% (105/158) who buy canned beans:**				
**Preferred canned bean traits**				
Price *	44.8	20.0 ^a^	41.0 ^a,b^	58.7 ^b^
Tradition—always buy type *	19	20.0 ^a^	30.8 ^b^	8.7 ^b^
Quality	28.6	15	38.5	26.1
Taste of beans	41	35	43.6	41.3
Nutritional value	22.9	5	23.1	30.4
Brand	21	1.9	9.5	9.5

* p < 0.05; ** *p* < 0.01; *** *p* < 0.001; Values with the same superscript letters are not significantly different.

**Table 3:** Attitudes and beliefs regarding bean consumption among low-income Iowa women by acculturation and ethnicity categories (*n* = 158; %).

Here Are Some Reasons People Have Said Prevent Them from Eating or Cooking Beans. Do You?	Strongly Disagree	Disagree	Agree	Strongly Agree	Do not Know
	%				
**1. Do not like taste of beans**	**51.9**	**27.8**	**9.5**	**7.6**	**3.2**
Hispanic-dominant	64.1	15.4	12.8	2.6	5.1
Bicultural/English-dominant	49.1	34	1.9	11.3	3.8
Non-Hispanic White	47	30.3	13.6	7.6	1.5
2. Family/children will not eat *	**54.2**	**29.7**	**8.4**	**4.5**	**3.2**
Hispanic-dominant	64.1 ^a^	30.8 ^a^	2.6 ^a^	2.6 ^a^	0 ^a^
Bicultural/English-dominant	60.8^a^	31.4 ^a,b^	5.9 ^a,b^	0 ^b^	2.0 ^a,b^
Non-Hispanic White	43.1^a^	27.7 ^a,b^	13.8 ^b,c^	9.2^c^	6.2 ^b,c^
**3. Beans cause intestinal gas**	**19.6**	**20.9**	**39.2**	**7.2**	**13.1**
Hispanic-dominant	26.3	10.5	26.3	13.2	23.7
Bicultural/English-dominant	22. 0	22	40	4	12
Non-Hispanic White	13.8	26.2	46.2	6.2	7.7
**4. Only poor people eat beans**	**71.6**	**17.4**	**3.9**	**1.3**	**5.8**
Hispanic-dominant	69.2	15.4	5.1	0	10.3
Bicultural/English-dominant	80	12	6	2	0
Non-Hispanic White	66.7	22.7	1.5	1.5	7.6
**5. Dry beans take too long to cook**	**26.5**	**22.6**	**29.7**	**14.2**	**7.1**
Hispanic-dominant	46.2	17.9	15.4	12.8	7.7
Bicultural/English-dominant	23.1	25	34.6	13.5	3.8
Non-Hispanic White	17.2	23.4	34.4	15.6	9.4
6. Difficult to make food with beans *	**53.9**	**36.4**	**3.9**	**0.6**	**5.2**
Hispanic-dominant	73.7 ^a^	21.1 ^b^	0 ^a,b^	0 ^a,b^	5.3 ^a,b^
Bicultural/English-dominant	57.7 ^a^	34.6 ^a^	1.9 ^a^	1.9 ^a^	3.8 ^a^
Non-Hispanic White	39.1 ^a^	46.9 ^b^	7.8 ^b^	0 ^b^	6.3 ^a,b^
**7. Beans not eaten by friends**	**40.1**	**38.2**	**3.8**	**1.3**	**16.6**
Hispanic-dominant	50	18.4	2.6	0	28.9
Bicultural/English-dominant	39.6	49.1	1.9	1.9	7.5
Non-Hispanic White	34.8	40.9	6.1	1.5	16.7
**Summary scale of 1** **–7 ^†^ (*n* = 149)**	**Total**	**Hispanic-dominant**	**Bicultural/English-dominant**	**Non-Hispanic White**	**Range**
Bean attitude scale (μ ± SD)	21.0 ± 5.1	21.3 ± 5.8	22.0 ± 4.1	20.0 ± 5.3	0 to 28

Numbers in bold represent the total percentages for all cases for each statement; * *p* < 0.05; ^†^ Questions were reverse-coded for summation; Values with the same superscript letters are not significantly different.

**Table 4:** Attitudes and beliefs about canned beans among low-income Iowa women by Bidimensional Acculturation Scale category (*n* = 158; %).

Here Are Some Reasons People Have Said Prevent Them from Eating or Cooking Beans. Do You?	Strongly Disagree	Disagree	Agree	Strongly Agree	Do not Know
	%				
1. Canned beans do not taste good ***	**29.1**	**40.4**	**16.6**	**6.6**	**7.3**
Hispanic-dominant	25.7 ^a,b^	14.3 ^b^	37.1 ^c^	11.4 ^a,c^	11.4 ^a,c^
Bicultural/English-dominant	19.6 ^a^	58.8 ^b^	9.8 ^a^	5.9 ^a,b^	5.9 ^a,b^
Non-Hispanic White	38.5 ^a^	40.0 ^a,b^	10.8 ^b^	4.6 ^a,b^	6.2 ^a,b^
2. Family will not eat canned beans ***	**41.8**	**32.3**	**10.8**	**8.2**	**7**
Hispanic-dominant	30.8 ^a,b^	15.4 ^b^	15.4 ^a,c^	25.6 ^d^	12.8 ^c,d^
Bicultural/English-dominant	49.1 ^a^	34.0 ^a,b^	11.3 ^a,b^	1.9 ^b^	3.8 ^a,b^
Non-Hispanic White	42.4 ^a,b^	40.9 ^b^	7.6 ^a,b^	3.0 ^a^	6.1 ^a,b^
**3. If I cannot cook beans myself will go without**	**37.4**	**36.1**	**15.5**	**5.2**	**5.8**
Hispanic-dominant	35.9	25.6	15.4	10.3	12.8
Bicultural/English-dominant	33.3	33.3	23.5	5.9	3.9
Non-Hispanic White	41.5	44.6	9.2	1.5	3.1
4. Canned beans are not true to culture **	**50**	**32.5**	**7.8**	**2.6**	**7.1**
Hispanic-dominant	38.9 ^a^	19.4 ^a^	16.7 ^b^	8.3 ^b^	16.7 ^b^
Bicultural/English-dominant	49.1 ^a^	35.8 ^a^	9.4 ^a^	1.9 ^a^	3.8 ^a^
Non-Hispanic White	56.9 ^a^	36.9 ^a^	1.5 ^b^	0 ^a,b^	4.6 ^a,b^
5. Canned beans are not healthy **	**32.7**	**27.6**	**10.9**	**7.7**	**21.2**
Hispanic-dominant	27.0 ^a,b^	8.1 ^b^	16.2 ^a.c^	16.2 ^c^	32.4 ^a,c^
Bicultural/English-dominant	24.5 ^a^	35.8 ^a^	13.2 ^a^	3.8 ^a^	22.6 ^a^
Non-Hispanic White	42.4 ^a^	31.8 ^a,b^	6.1 ^b^	6.1 ^a,b^	13.6 ^b^
**6. Canned beans are too expensive**	**36.1**	**39.9**	**10.1**	**1.9**	**12**
Hispanic-dominant	30.8	25.6	20.5	2.6	20.5
Bicultural/English-dominant	34	47.2	5.7	1.9	11.3
Non-Hispanic White	40.9	42.4	7.6	1.5	7.6
**7. Canned beans have preservatives**	**11.4**	**10.1**	**35.4**	**13.3**	**29.7**
Hispanic-dominant	10.3	0	35.9	23.1	30.8
Bicultural/English-dominant	13.2	7.5	39.6	7.5	32.1
Non-Hispanic White	10.6	18.2	31.8	12.1	27.3
**Summary scale of questions 1–7 ^†^**	**Total**	**Hispanic-dominant**	**Bicultural/English-dominant**	**Non-Hispanic White**	**Range**
Canned bean attitude scale ***	18.8 ± 6.1	15.5± 7.0	19.1 ± 5.0	20.4 ± 5.8	0 to 28
(*n* = 153; μ ± SD)					

Numbers in bold represent the total percentages for all cases for each statement; ** *p* < 0.01; *** *p* < 0.001; ^†^ “Do not know” coded as 0 for summation; Values with the same superscript letters are not significantly different. SD, Standard Deviation.
